# Inhibiting autophagy selectively prunes dysfunctional tumor vessels and optimizes the tumor immune microenvironment

**DOI:** 10.7150/thno.98285

**Published:** 2025-01-01

**Authors:** Wanting Hou, Chaoxin Xiao, Ruihan Zhou, Xiaohong Yao, Qin Chen, Tongtong Xu, Fujun Cao, Yulin Wang, Xiaoying Li, Ouying Yan, Xiaolin Ai, Cheng Yi, Dan Cao, Chengjian Zhao

**Affiliations:** 1 Department of Abdominal Oncology, West China Hospital, Sichuan University, Chengdu, 610041, Sichuan Province, People's Republic of China.; 2 State Key Laboratory of Biotherapy and Cancer Center, West China Hospital, Sichuan University, and Collaborative Innovation Center for Biotherapy, Chengdu, Sichuan Province, People's Republic of China.; 3 Department of Pathology, West China Second University Hospital, Sichuan University, Chengdu, 610041, Sichuan Province, People's Republic of China.

**Keywords:** tumor endothelial cell, autophagy, dysfunctional tumor vessels, tumor immune microenvironment, tumor therapy

## Abstract

Dysfunctional tumor vasculature, hypoxia, and an immunosuppressive microenvironment are significant barriers to effective cancer therapy. Autophagy, which is critical for maintaining cellular homeostasis and apoptosis resistance, is primarily triggered by hypoxia and nutrient deprivation, conditions prevalent in dysfunctional tumor vessels due to poor circulation. However, the role of autophagy in dysfunctional tumor endothelial cells and its impact on treatment and the tumor microenvironment (TME) remain poorly understood.

**Methods:** We used multiplex immunofluorescence and transgene-based imaging to characterize autophagy in endothelial cells from clinical tumor samples, zebrafish xenograft tumors, and murine models. Using a zebrafish xenograft vasculature platform, we analyzed the effects of autophagy inhibitors on the structure and function of the tumor vasculature. In mice, we investigated autophagy inhibition via endothelial-specific autophagy gene knockout (*Atg7*^iECKO^) and the autophagy inhibitor SBI-0206965 and evaluated the synergistic effects of combining SBI-0206965 with low-dose chemotherapy (5-fluorouracil, 5-FU) or PD-1 antibody. Human umbilical vein endothelial cells (HUVECs) were cultured *in vitro* under hypoxic, glucose-deprived, and serum-free conditions to simulate dysfunctional tumor endothelial cells and to explore the mechanisms by which autophagy inhibition optimizes tumor vasculature.

**Results:** Elevated autophagy was observed in tumor endothelial cells within the dysfunctional vasculature. Autophagy inhibition, through either genetic knockout or pharmacological inhibition, selectively prunes dysfunctional vessels and improves vascular function. It also stimulates the formation of a perivascular immune niche, thereby optimizing the tumor immune microenvironment (TiME). Furthermore, combining the autophagy inhibitor SBI-0206965 with low-dose 5-FU or PD-1 antibody potentiated the anti-tumor effects. Mechanistic studies have indicated that autophagy acts as a protective response to the hypoxic and nutrient-deprived TME, while its inhibition, mediated by p53 activation, promotes tumor endothelial cell apoptosis in dysfunctional tumor vessels, further optimizing the structure and function of the tumor vasculature.

**Conclusions:** Targeting endothelial cell autophagy is a promising strategy for remodeling the dysfunctional tumor vasculature, optimizing the TiME, and boosting the efficacy of chemotherapy and immunotherapy.

## Introduction

Solid tumor growth is accompanied by the formation of an aberrant tumor vascular network. Within the tumor microenvironment (TME), various cellular components secrete a substantial quantity of pro-angiogenic factors, driving the rapid development of tumor blood vessels to meet the increasing demand for oxygen and nutrients essential for tumor progression. However, unlike normal tissue vasculature, tumor vessels exhibit immature and dysfunctional structures 1. Previous studies have demonstrated a significant presence of dysfunctional vascular sprouts within the aberrant tumor vasculature, which play a pivotal role in maintaining the abnormal and disorganized vascular structure 2, 3. These dysfunctional tumor vascular sprouts not only support the formation of abnormal vasculature but also contribute to tumor hypoxia and an immunosuppressive TME, factors closely linked to malignant characteristics such as tumor progression, metastasis, radioresistance, chemoresistance, and immune evasion. Abnormal tumor vasculature also severely limits tumor blood perfusion, impairing the delivery and penetration of chemotherapeutic and immunotherapeutic agents and restricting immune cell infiltration, thereby undermining the effectiveness of anti-tumor therapies 4.

Recent studies have highlighted the importance of optimizing tumor vasculature in solid tumors to improve overall vascular structure and function, thereby ameliorating tumor hypoxia and the immunosuppressive TME. Tumor vascular optimization has shown significant potential for enhancing the delivery of anti-cancer drugs and immune cells, ultimately leading to synergistic improvements in the efficacy of chemotherapy and immunotherapy 5-7. Current clinical and preclinical studies have predominantly focused on anti-angiogenic therapies that target the VEGF-VEGFR pathway to modulate the balance between pro- and anti-angiogenic factors, thereby inducing tumor vascular normalization 8, 9. However, this approach relies on a short-lived window of balance, which results in a transient and unstable normalization effect 10. Moreover, long-term use of anti-angiogenic agents has been associated with severe adverse effects in patients 11. Therefore, it is imperative to explore novel and more effective strategies for optimising the structure and function of tumour vasculature.

In this study, we propose selective pruning of dysfunctional tumor vessels as a novel approach to optimize the overall structure and function of tumor vasculature. We focused on endothelial cell autophagy, a fundamental homeostatic process in eukaryotic cells that plays a key role in resisting apoptosis induced by adverse environmental conditions 12. Hypoxia and nutrient deprivation are critical inducers of autophagy. Within tumors, both tumor and stromal cells utilize autophagy to survive in the harsh TME, counteracting hypoxia induced by hypoxia and nutrient deprivation. Additionally, autophagy plays a crucial role in maintaining an immunosuppressive TME 13. Tumor endothelial cells (TECs) are the primary structural cells of tumor blood vessels and also serve as key components of the tumor stroma. TECs are constantly exposed to pro-angiogenic signals, resulting in functional and structural abnormalities 14. A previous study demonstrated that TECs are more prone to autophagy, but less likely to undergo apoptosis 15. Moreover, recent research on melanoma has identified TEC autophagy as a key immune checkpoint, suggesting that it plays a critical role in immune evasion 16. Autophagy in endothelial cells has been implicated in angiogenesis under various pathological conditions 17. It may serve as a crucial survival mechanism for endothelial cells in dysfunctional tumor vessels, which are particularly affected by severe hypoxia and nutrient deprivation due to limited blood flow. However, current research lacks a detailed understanding of the autophagic characteristics of endothelial cells in dysfunctional tumor vessels and their specific roles in tumor vasculature and immune regulation. Understanding these processes could provide novel insights into targeting tumor angiogenesis and offer potential strategies for modulating immune responses in cancer therapy.

In this study, we investigated the autophagic properties of endothelial cells in dysfunctional tumor vessels and explored the impact of autophagy on the remodeling of abnormal tumor vasculature and optimization of the tumor immune microenvironment (TiME). Our findings provide a foundation for developing innovative therapeutic strategies targeting TECs to optimize the tumor vasculature and enhance the overall therapeutic efficacy of cancer treatments.

## Results

### TECs exhibit elevated autophagy levels

**Figure 1:**
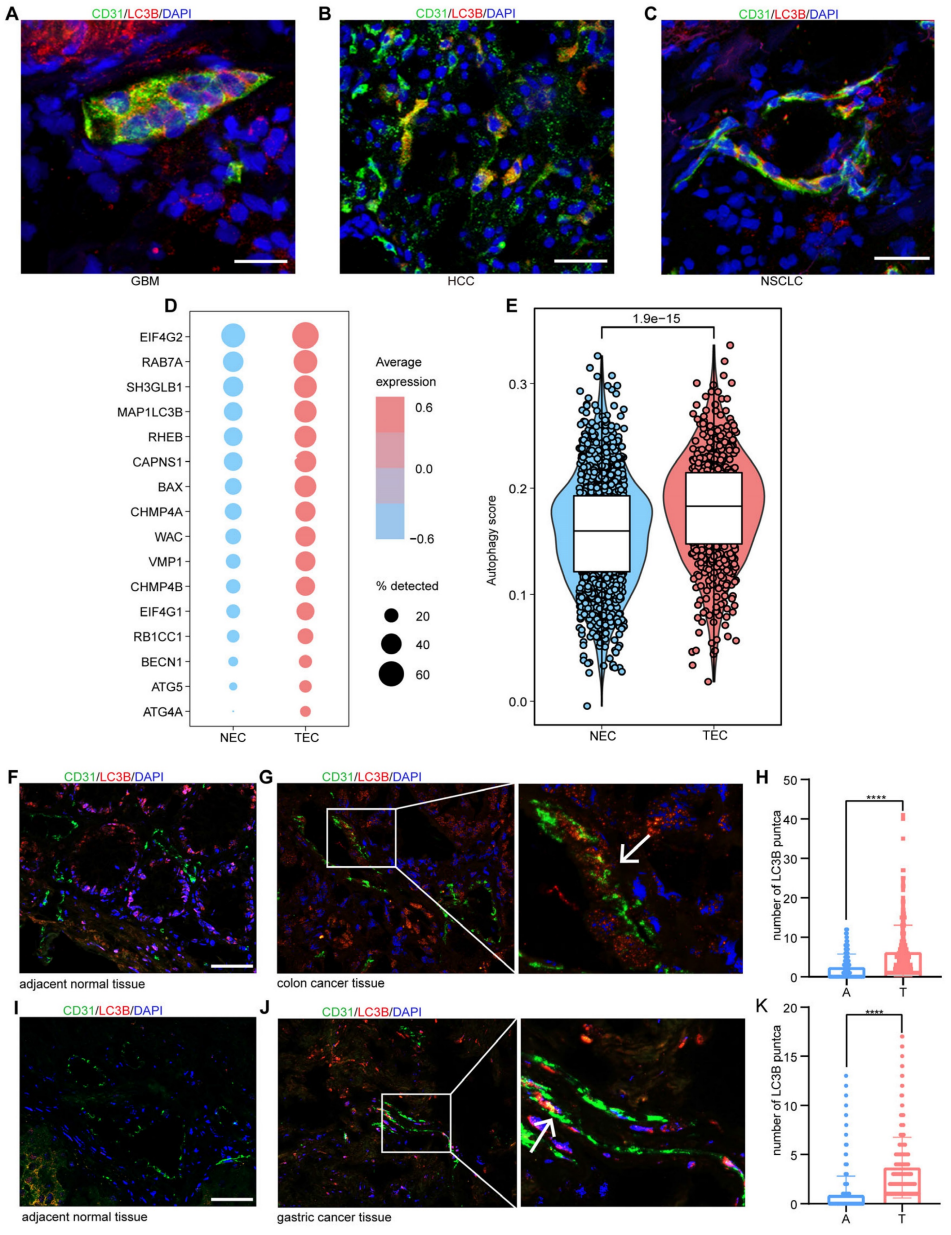
** TECs exhibit elevated autophagy levels.** A-C: Representative immunofluorescence images showing the expression of autophagy-related protein LC3B (red) in CD31^+^ vessels (green) in tumor tissues from patients with hepatocellular carcinoma (HCC), glioblastoma (GBM), and non-small cell lung cancer (NSCLC). Scale bars = 20 μm. *n* = 3 patients per group. D: Dot plot visualization of autophagy-related gene expression in NECs and TECs. The x-axis represents the tissue source (NEC or TEC), whereas the y-axis lists the autophagy-related genes. Dot size indicates the percentage of samples in which each gene was detected, and dot color represents the average gene expression level across samples. The color scale ranges from blue (low expression) to red (high expression). E: Comparison of autophagy gene expression scores between NECs and TECs, revealing a statistically significant increase in autophagy-related gene expression in TECs (*P*-value = 1.9e-15). F-K: Representative immunofluorescence images showing LC3B (red) in endothelial cells (indicated by arrows) within both adjacent normal and tumor tissues from patients with colon and gastric cancer. Box plots (H and K) quantifying the number of LC3B-positive puncta in CD31^+^ cells from adjacent normal (A) and tumor (T) tissues in colon and gastric cancer samples. Box plots display the maximum and minimum values, medians, and 25th/75th percentiles. *P* values were calculated using two-tailed Student's t-tests. ****P < 0.0001. Scale bars = 50 μm. *n* = 3 patients per group.

We examined surgical specimens from various solid tumors to assess the autophagic features of the tumor endothelium. Immunofluorescence (IF) staining revealed the expression of autophagy-related protein LC3B in tumor vascular endothelial cells across several cancer types, including hepatocellular carcinoma (HCC), non-small cell lung cancer (NSCLC), glioblastoma (GBM), gastric cancer (GC), and colorectal cancer (CRC). We observed a significant accumulation of LC3B-positive puncta within the endothelial cells of these tumors, indicating heightened autophagic activity and suggesting an active role for autophagy in tumor endothelial cell homeostasis (Figure 1A-C, G, J). In addition, we employed multiplex immunofluorescence (mIF) staining to detect the expression of key autophagic markers, including Beclin-1, p62, and LC3B, in intrahepatic cholangiocarcinoma (ICC) tumor vasculature. Our results demonstrated LC3B and Beclin-1 expression in the tumor vasculature, while p62 was not expressed (Figure S1). These results collectively confirm the activation of autophagy pathways within the tumor vasculature, as p62 plays a role in autophagic degradation.

We further analyzed the expression of autophagy-related gene signatures in both normal endothelial cells (NECs) and TECs using single-cell RNA sequencing (scRNA-seq) data from NSCLC, as reported previously 18. Consistent with our IF results, TECs exhibited significantly higher expression of autophagy-related genes than normal lung endothelial cells (Figure 1D-E). These observations were further validated in clinical samples from patients with colon and gastric cancers, where a pronounced increase in autophagic puncta within TECs was observed (Figure 1F-K). Collectively, these findings suggest that enhanced autophagic activity is a hallmark of TECs in multiple tumor types.

### Endothelial autophagy is preferentially induced in dysfunctional tumor vessels

**Figure 2:**
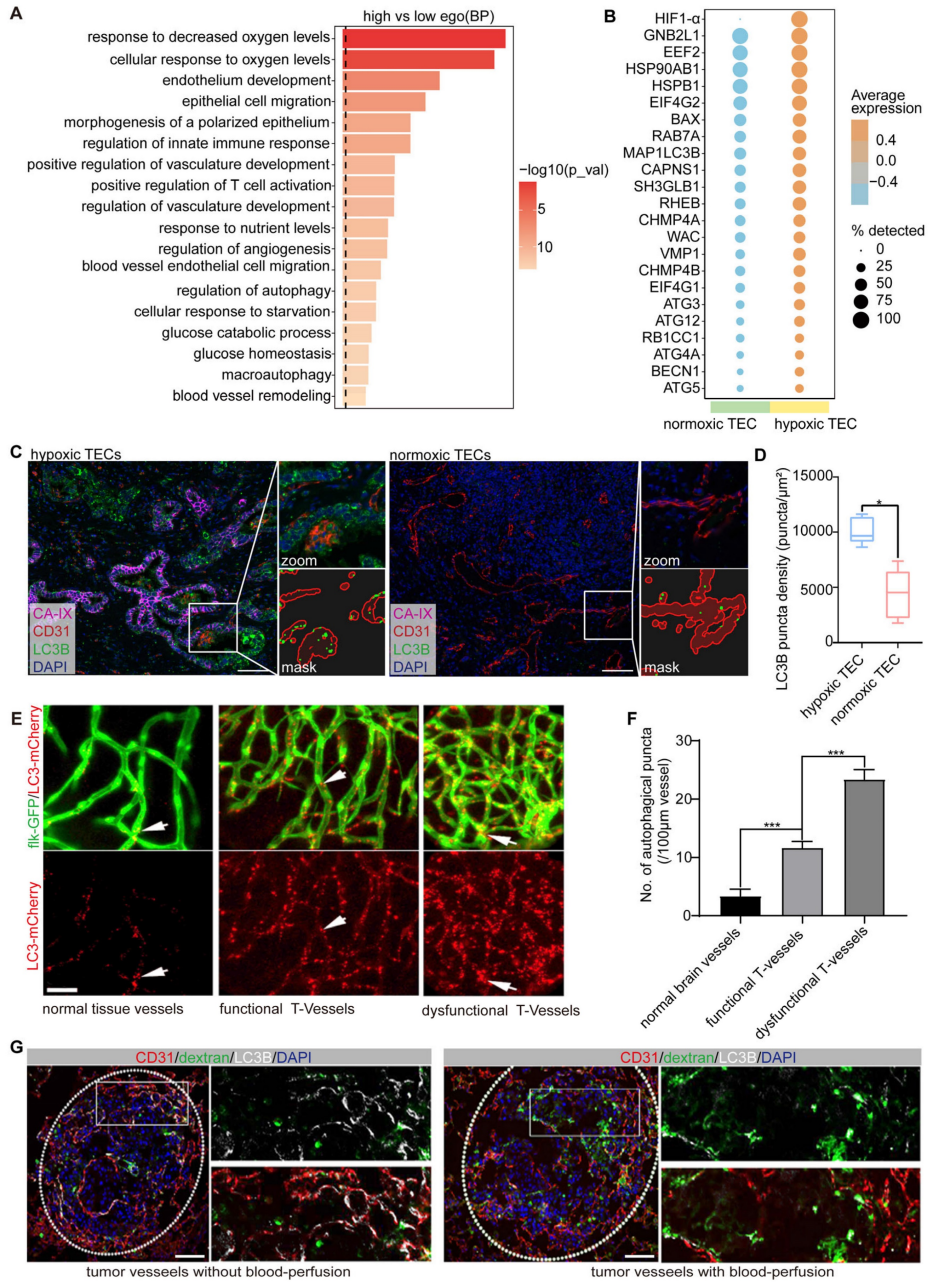
** Endothelial autophagy is preferentially induced in dysfunctional tumor vessels.** A: Pathway enrichment analysis based on the DEGs (top 500 highly expressed genes) in endothelial cells with high- versus low-autophagy gene scores. B: Dot plot comparing the expression of autophagy-related genes in normoxic (HIF-1α-negative) versus hypoxic (HIF-1α-positive) TECs. The color scale indicates expression levels, whereas dot size reflects the percentage of samples in which the gene was detected. C and D: Multiplex immunofluorescence analysis of autophagic activity in the vasculature of hypoxic and normoxic regions in ICC tissues. CA-IX (magenta) labels hypoxic regions, CD31 (red) identifies endothelial cells in the tumor vasculature, and LC3B (green) indicates autophagic activity. The zoomed-in section highlights a specific region comparing LC3B density in the tumor vasculature between the hypoxic and normoxic areas. The corresponding mask image shows the precise localization of the LC3B puncta (autophagic vesicles) within the zoomed region. The right panel (D) presents a box plot quantifying LC3B puncta density (puncta/μm²) in TECs from hypoxic and normoxic regions, showing significantly higher autophagic activity in hypoxic areas (*P < 0.05). Scale bar = 100 μm. E and F: *In vivo* 3D imaging of autophagy in the zebrafish tumor xenograft vasculature. Flk-GFP (green) visualizes the vessel structure, while LC3-mCherry (red) indicates autophagy in normal brain vessels, as well as functional tumor vessels and dysfunctional tumor vessels in CT26 cells at 4 dpi. Quantification of autophagic puncta showed a significant increase in dysfunctional tumor vessels compared to that in normal and functional vessels (***P < 0.001). Scale bar = 50 μm. G: Discrimination between autophagic and perfused endothelia in B16-F10 melanoma mouse tumor xenografts. Double immunostaining for CD31 (red) and LC3B (white) was used to label the endothelial cells and autophagy, respectively, with DAPI (blue) counterstaining of the nuclei. Perfused vessels were detected using fluorescein isothiocyanate (FITC)-dextran angiography (green). Autophagic endothelium (LC3B^+^ CD31^+^) was predominantly observed in non-perfused tumor vessels, with minimal overlap between FITC-dextran-positive (perfused) vessels and autophagic cells. Scale bar = 100 μm.

Using scRNA-seq data from patients with NSCLC 18, we analyzed the expression profiles of 245 autophagy-related genes in TECs. TECs were categorized into high-autophagy and low-autophagy subpopulations based on their autophagic activity (Figure S2). Pathway enrichment analysis of the top 500 differentially expressed genes (DEGs) revealed that autophagy-high TECs were significantly enriched in pathways associated with the hypoxia response, endothelial development, morphogenesis, and cell migration (Figure 2A). These findings suggest a strong correlation between elevated autophagy in TECs and processes such as hypoxia and vascular remodeling.

To further investigate this relationship, we used hypoxia-inducible factor 1-alpha (HIF-1α) as a marker to distinguish between hypoxic TECs from normoxic TECs. Our analysis demonstrated that autophagy-related genes, including *MAP1LC3B*, *BECN1*, *ATG5*, *ATG12*, *ATG4A*, *RAB7A*, and *EIF4G1*, were significantly upregulated in HIF-1α-positive TECs compared to HIF-1α-negative TECs (Figure 2B). This upregulation underscores the robust association between hypoxia and increased autophagic activity in TECs. We validated these findings using clinical samples from ICC, where mIF staining for CA-IX (a hypoxia marker), LC3B, and CD31 revealed significantly higher autophagic activity in endothelial cells located in hypoxic regions compared to normoxic regions (Figure 2C-D). These results strongly indicated that hypoxia is closely associated with elevated autophagy in TECs.

To investigate the spatial distribution and dynamics of autophagy-positive endothelial cells *in vivo*, we used a transgenic zebrafish model (flk-GFP/LC3-mCherry) to establish a tumor xenograft using CT26 colon carcinoma cells, as previously described 3. Our previous study demonstrated that functional tumor vasculature begins to form between 4 and 5 d post-implantation (dpi). Accordingly, we conducted *in vivo* 3D imaging of the entire tumor vasculature in zebrafish xenografts at 4 dpi (*n* = 20). Notably, we observed extensive LC3-mCherry aggregates within the tumor endothelium during vascularization, which were markedly distinct from those in normal tissues (Figure 2E-F). These aggregates were predominantly concentrated in endothelial cords lacking vessel lumens, indicating that autophagy is closely linked to vascular dysfunction in these regions.

We also assessed blood perfusion and autophagic activity in the tumor endothelium using a B16-F10 melanoma mouse xenograft model. B16-F10 xenografts were established via intravenous injection of 50,000 cells per mouse (*n* = 5), and the tumor vasculature was evaluated at 12 dpi. Blood perfusion was assessed using fluorescein isothiocyanate-dextran angiography, and the autophagic endothelium was identified by double immunostaining for CD31 and LC3B. Consistent with our findings in human tumor samples and zebrafish xenografts, autophagic endothelium (LC3B^+^ CD31^+^) was consistently present but not uniformly distributed throughout the tumor vasculature. Moreover, the alignment of immunofluorescence images with FITC-dextran angiography revealed that the autophagic endothelium (LC3B^+^ CD31^+^) and functional endothelium (FITC^+^) were mutually exclusive (Figure 2G), indicating that autophagy predominantly occurs in non-perfused, dysfunctional vessels.

In conclusion, our results in zebrafish, human, and mouse models collectively demonstrate that endothelial autophagy is preferentially induced in dysfunctional tumor vessels characterized by poor blood flow and hypoxia.

### Autophagy inhibitors optimize tumor vascular network functionality by targeting dysfunctional tumor vessels

**Figure 3:**
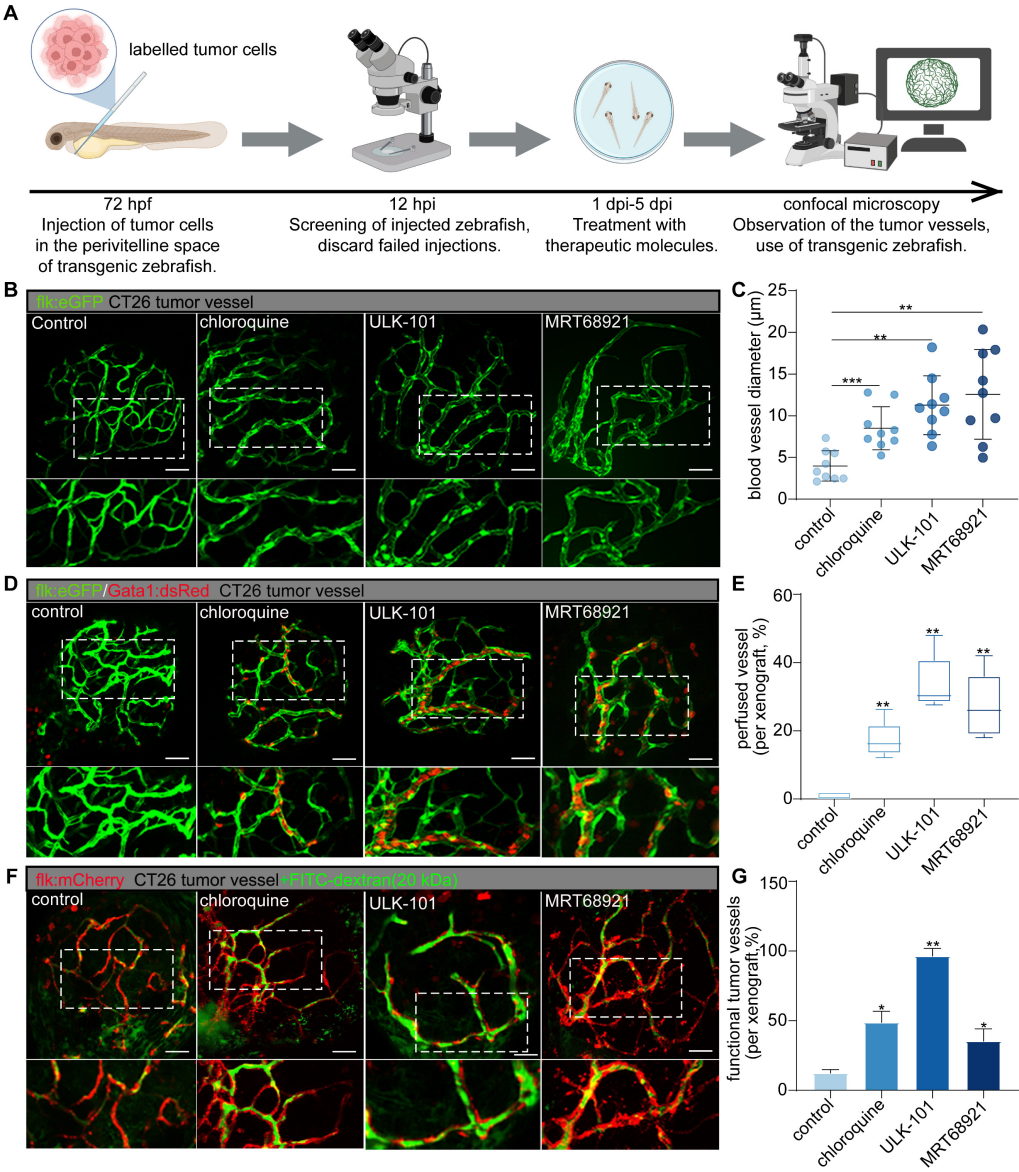
** Autophagy inhibitors can selectively prune dysfunctional tumor vessels, optimizing the structure and function of the tumor's vascular network.** A: Experimental timeline for evaluating the effects of autophagy inhibitors on zebrafish CT26 tumor vasculature. Fluorescently labelled tumor cells were injected into zebrafish 72 h post-fertilization (hpf), followed by screening 12 h post-injection (hpi). Treatment with autophagy inhibitors (chloroquine, 50 μmol/L; ULK-101, 4 μmol/L; MRT68921, 50 μmol/L) was administered from 1 d post-injection (dpi) to 5 dpi, with subsequent assessment of tumor vessels using confocal microscopy. B and C: Confocal 3D projection images of CT26 tumor vessels in the control and autophagy inhibitor-treated groups, with vessels highlighted in green. Blood vessel diameters were quantified using ImageJ software. Quantitative graph showing blood vessel diameter comparisons among the different groups (C). Quantitative data are shown as the mean ± SD. D and E: Confocal images of transgenic zebrafish expressing the endothelial marker flk:GFP and erythrocyte marker Gata1:dsRed in CT26 tumor vessels for the control and autophagy inhibitor-treated groups. Boxplot graph showing the persistence of blood flow quantified as a percentage of the luminated area. Boxplot showing the maximum and minimum values, medians, and 25/75 percentiles. F and G: Confocal images showing injection of low-molecular-weight FITC-dextran (20 kDa) in CT26 tumor vessels for the control and autophagy inhibitor-treated groups, with tumor vessels labelled in red and dextran in green. Bar graph depicting FITC signal intensity within tumor vessels. Quantitative data are shown as the mean ± SD. *P* values (versus control) were generated using one-way analysis of variance, followed by Tukey's post-hoc tests for multiple comparisons. *P < 0.05, **P < 0.01, ***P < 0.001. Scale bars = 50 μm.

To investigate the role of endothelial autophagy inhibition in optimizing tumor vasculature, we employed small-molecule autophagy inhibitors (chloroquine, ULK-101, and MRT-68921) using our previously established zebrafish functional xenograft vasculature platform (zFXVP) with CT26 tumor cell xenografts 3. This model allowed us to visualize the impact of autophagy inhibition on both the tumor vascular structure and function. Chloroquine targets late-stage autophagy by preventing autophagosome-lysosome fusion, whereas ULK-101 and MRT-68921 inhibit autophagy initiation by targeting ULK1. This enabled us to investigate the effects of both early and late autophagy inhibition on tumor vasculature. Treatment with autophagy inhibitors began at 1 dpi and continued until 5 dpi, coinciding with tumor vasculature formation in CT26 xenografts (Figure 3A).

Confocal 3D projection imaging revealed a significant and consistent increase in tumor vessel diameter in the groups treated with autophagy inhibitors compared to that in the control group (Figure 3B-C). This increase in vessel diameter was accompanied by a higher proportion of lumenized vessels in the treated groups, suggesting enhanced blood perfusion. Additionally, there was a notable reduction in the number of dysfunctional tumor vessels (Figure 3B-G).

To comprehensively assess tumor vessel functionality following autophagy inhibition, we used double-transgenic zebrafish (flk:GFP/Gata1:dsRED) and performed angiography using low-molecular-weight FITC-dextran (20 kDa). In the autophagy inhibitor-treated zebrafish, sustained blood flow was observed at 5 dpi, in contrast to the control group (Figure 3D-E). Furthermore, the injection of FITC-dextran into intersegmental vessels resulted in significantly higher intravascular FITC signal intensity in the autophagy inhibitor-treated xenografts than in the controls, along with reduced leakage of FITC into the tumor mass (Figure 3F-G).

Collectively, these findings suggest that the inhibition of autophagy during tumor vascularization enhances the functionality and maturation of new blood vessels within the TME. Using this visualization model, we observed that autophagy inhibition selectively pruned the dysfunctional tumor vessels, thereby optimizing the overall structure and function of the tumor vascular network. Both early-stage autophagy inhibitors (ULK-101 and MRT-68921) and a late-stage inhibitor (chloroquine) effectively promoted the selective pruning of dysfunctional tumor vessels. Notably, ULK-101 and MRT-68921 exhibited superior efficacy compared to chloroquine in optimizing tumor vasculature (Figure 3B-G). Autophagy inhibitors enhance blood perfusion and vessel integrity by selectively targeting dysfunctional tumor vessels, thereby improving tumor vascular functionality.

### Blocking endothelial autophagy optimizes tumor vasculature and enhances the TiME

**Figure 4:**
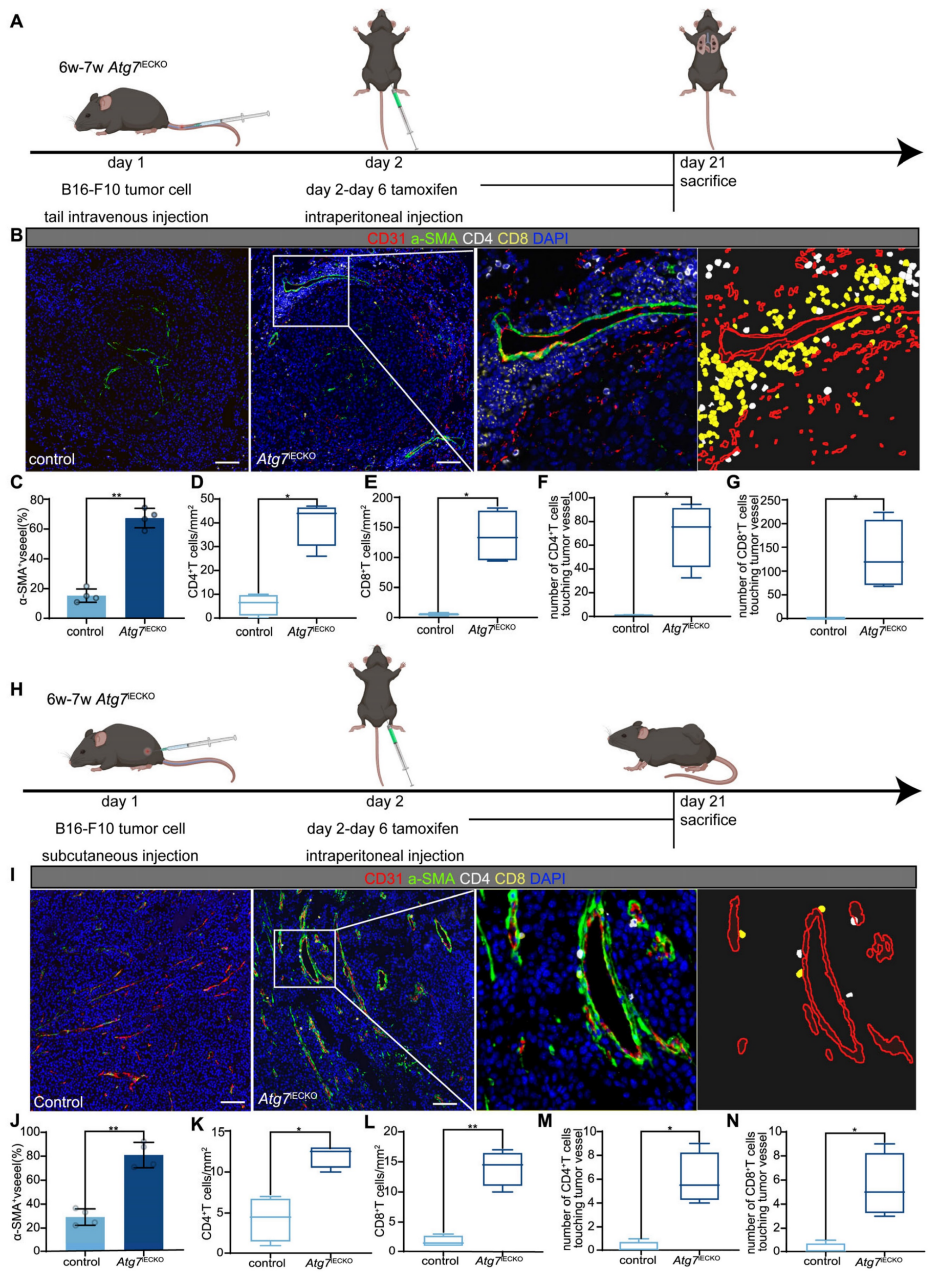
** Endothelial autophagy blockade remodels tumor vasculature and enhances perivascular immune niche formation in *Atg7*^iECKO^ mice.** A: Schematic of the experimental timeline for the B16-F10 lung metastasis model in *Atg7*^iECKO^ mice. On day 2 after the intravenous injection of B16-F10 cells, tamoxifen (20 mg/ml in corn oil) was administered intraperitoneally (2 mg/20 g body weight) for five consecutive days to induce endothelial cell-specific *Atg7* knockout. Tumor tissues were harvested 21 d post-transplantation. B: Representative mIF images of tumor sections from control and *Atg7*^iECKO^ mice, stained for CD31 (endothelial cells, red), α-SMA (pericytes, green), CD4 (helper T cells, white), CD8 (cytotoxic T cells, yellow), and DAPI (nuclei, blue). Right panel: Enlarged view showing T cell aggregates in the perivascular immune niche of *Atg7*^iECKO^ tumor tissue. Scale bar = 100 μm. C: Quantification of functional tumor vessels, defined as the percentage of CD31^+^ vessels co-stained with α-SMA. Data are presented as the mean ± SD (*n* = 4 mice per group; **P < 0.01, Student's t-test). D-G: Quantification of CD4^+^ and CD8^+^ T cell density in the tumor stroma and the number of these cells within a 10-μm radius around tumor blood vessels. Data are shown as box plots displaying the minimum, maximum, median, and 25/75 percentiles (*n* = 4 mice per group; *P < 0.05, **P < 0.01, Student's t-test). H: Schematic of the experimental timeline for the B16-F10 subcutaneous tumor model in *Atg7*^iECKO^ mice. On day 2 following the subcutaneous injection of B16-F10 cells, tamoxifen (20 mg/ml in corn oil) was administered intraperitoneally (2 mg/20 g body weight) for five consecutive days to induce endothelial cell-specific *Atg7* knockout. Tumor tissues were collected on day 21 post-transplantation. I: Representative mIF images of tumor sections from control and *Atg7*^iECKO^ mice, stained for CD31 (endothelial cells, red), α-SMA (pericytes, green), CD4 (helper T cells, white), CD8 (cytotoxic T cells, yellow), and DAPI (nuclei, blue). Right panel: Enlarged view showing T cell aggregates in the perivascular immune niche of *Atg7*^iECKO^ tumor tissue. Scale bar = 100 μm. J: Quantification of functional tumor vessels, defined as the percentage of CD31^+^ vessels co-stained with α-SMA. Data are presented as the mean ± SD (*n* = 4 mice per group; **P < 0.01, Student's t-test). K-N: Quantification of CD4^+^ and CD8^+^ T cell density in the tumor stroma and the number of these cells within a 10-μm radius around tumor blood vessels. Data are shown as box plots displaying the minimum, maximum, median, and 25/75 percentiles (*n* = 4 mice per group; *P < 0.05, **P < 0.01, Student's t-test).

To investigate the role of blocking endothelial autophagy in tumor vasculature and immune microenvironment optimization, we utilized endothelial cell-specific Atg7 conditional knockout (*Atg7*^iECKO^) mice generated by crossing VE-Cadherin-Cre-ERT2 transgenic mice with *Atg7*-floxed mice. In these *Atg7*^iECKO^ mice, we established B16-F10 subcutaneous tumor and lung metastasis models. Endothelial-specific Atg7 knockout was induced via tamoxifen administration, initiated the day after tumor inoculation, to ensure knockout during the formation of the tumor vascular network (Figure 4A, H). Genetic inhibition of endothelial autophagy in these mice led to remodeling of the aberrant tumor vasculature, resulting in a significant increase in functional tumor blood vessels and a marked elevation in the overall density of CD8^+^ and CD4^+^ T cells within the tumor stroma (Figure 4B-G and I-N). These findings indicate that blocking endothelial autophagy enhances the TiME. Notably, T cells tended to aggregate around functional tumor vessels, forming distinct perivascular immune niches (Figure 4B, I), which have been previously linked to improved responses to immunotherapy 19, 20.

To assess whether the pharmacological inhibition of autophagy could replicate the effects observed with genetic autophagy inhibition, we utilized a CT26 subcutaneous tumor model treated with SBI-0206965, a small-molecule ULK1 inhibitor whose safety has been well-documented in a previous mouse study 21. Drug treatment commenced on day 7 post-CT26 cell transplantation, following tumor size measurement and random assignment to treatment groups. Based on previously established safety data, SBI-0206965 was administered intraperitoneally once daily at 10 mg/kg 21. After 14 days of treatment, tumor tissues were harvested for analysis (Figure S3A). mIF staining revealed significant increases in functional T cells (CD4^+^ granzyme B^+^ and CD8^+^ granzyme B^+^ T cells) and structural changes in tumor vessels (CD31/aSMA staining) in the SBI-0206965-treated group. Consistent with the observations in* Atg7*^iECKO^ mice, pharmacological inhibition of autophagy resulted in increased T cell infiltration, particularly around mature tumor vessels characterized by large lumens and aSMA^+^ coverage, suggesting the formation of perivascular immune niches (Figure S3B-K).

These results suggest that blocking endothelial autophagy, either genetically or pharmacologically, improves tumor vasculature functionality and enhances the TiME, which may have therapeutic implications in synergy with immunotherapy.

### Enhancing anti-tumor efficacy of anti-PD-1 immunotherapy or chemotherapy with autophagy inhibitor SBI-0206965

Our findings demonstrate that inhibiting endothelial autophagy improves tumor vascular function and enhances T cell infiltration in the TME. This suggests that autophagy inhibition can synergize with the anti-tumor effects of immune checkpoint inhibitors or chemotherapy. Given that combination therapies involving vascular-targeting drugs with chemotherapy or immunotherapy are common in CRC treatment, we employed a CT26 colon carcinoma subcutaneous tumor model to evaluate the effects of combining the autophagy inhibitor SBI-0206965 with low-dose chemotherapy (5-fluorouracil, 5-FU) or anti-PD-1 monoclonal antibodies.

Initially, we assessed the anti-tumor efficacy of SBI-0206965 in combination with a low dose of 5-FU. We treated mice with 10 mg/kg of the autophagy inhibitor SBI-0206965, followed by administration of a low dose of 5-FU (5 mg/kg) (Figure S4A). The combined treatment significantly suppressed tumor growth compared to treatment with either drug alone (Figure S4K-L). We further analyzed the immune microenvironment using mIF (Figure S4B). The results showed that the group receiving the combination of SBI-0206965 and low-dose 5-FU exhibited a significant reduction in tumor vascular density compared with the chemotherapy-only group (Figure S4C). However, the proportion of functional vessels (CD31^+^ α-SMA^+^ vessels) was markedly increased (Figure S4D). Additionally, the densities of CD3^+^ T cells, CD8^+^ T cells, granzyme B^+^ cells, and CD8^+^ granzyme B^+^ cytotoxic T cells were significantly higher in the combination treatment group (Figure S4E-H). Notably, there was a substantial increase in the number of perivascular CD8^+^ T cells and CD8^+^ granzyme B^+^ cells (Figure S4I-J), which may have contributed to the enhanced anti-tumor efficacy observed with the combined treatment.

**Figure 5:**
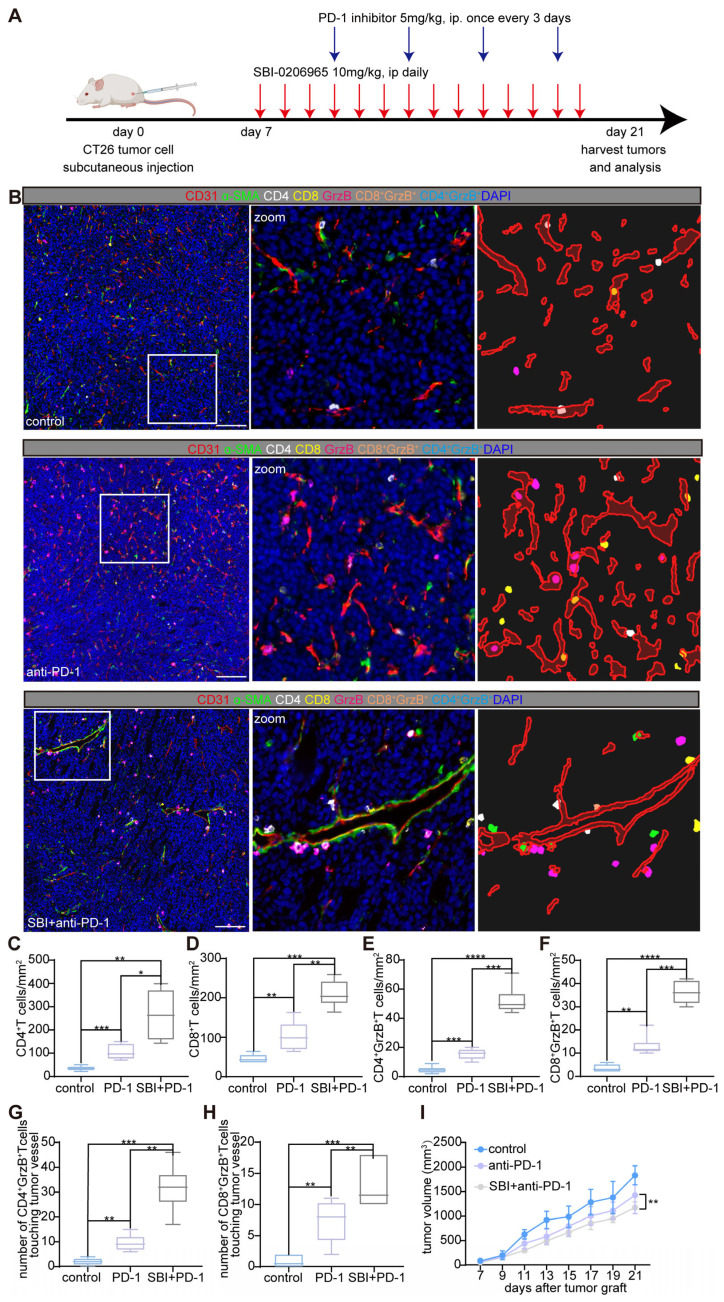
** Autophagy inhibitor (SBI-0206965) combined with anti-mouse PD-1 (CD279) induces perivascular immune niches formation and improves the TiME.** A: Schematic representation of the treatment regimen used in the CT26 murine subcutaneous tumor model. Mice were treated with the autophagy inhibitor SBI-0206965 (10 mg/kg, intraperitoneally, daily starting from day 7) and an anti-PD-1 antibody (5 mg/kg, intraperitoneally, every 3 d starting on day 11 post-tumor inoculation). Tumor tissues were harvested on day 21 for further analyzes. B: Representative mIF images of tumor sections from the control, PD-1 inhibitor-treated, and combination (SBI-0206965 + PD-1 inhibitor)-treated groups. TiME was stained for CD4^+^ helper T cells (white), CD8^+^ cytotoxic T cells (yellow), granzyme B^+^ cells (GrzB, magenta), endothelial cells (CD31, red), and pericytes (α-SMA, green). Nuclei were stained with DAPI (blue). The zoomed-in regions highlight immune cell distribution and formation of perivascular immune niches in the combination therapy group. Scale bars = 100 μm. C-F:Quantitative analysis of immune cell densities in the tumor stroma, including CD4^+^ T cells (C), CD8^+^ T cells (D), CD4^+^ granzyme B^+^ T cells (E), and CD8^+^ granzyme B^+^ T cells (F). G and H: Quantification of the number of CD4^+^ granzyme B^+^ T cells (G) and CD8^+^ granzyme B^+^ T cells (H) within a 10-μm radius around tumor vessels. Data are presented as box plots showing the maximum and minimum values, medians, and 25/75 percentiles (*n* = 7 mice per group; *P < 0.05, **P < 0.01, ***P < 0.001, ****P < 0.0001; One-way ANOVA). I: Tumor volume changes in each treatment group starting on the day of tumor grafting. Tumor growth was significantly reduced in the combination therapy group (**P < 0.01, two-way ANOVA with Bonferroni post-hoc test). Data are represented as the mean ± SEM.

To explore the potential synergistic effects of autophagy inhibition and immune checkpoint blockade, we initiated combination treatment with SBI-0206965 and an anti-mouse PD-1 (CD279) antibody in a CT26 subcutaneous tumor model. Starting on day 7 post-tumor inoculation, the mice received 10 mg/kg SBI-0206965 daily via intraperitoneal injection. Four days after the initiation of SBI-0206965 treatment, anti-PD-1 antibody was administered intraperitoneally at a dose of 5 mg/kg every 3 d. The tumors were harvested on day 21 for volumetric and weight analyzes (Figure 5A). Comprehensive analysis of the TME and tumor vasculature using mIF (Figure 5B) revealed a moderate synergistic anti-tumor response in the combination therapy group, as shown in Figure 5I.

Immunophenotyping of the tumors showed a marked increase in the infiltration of CD4^+^ and CD8^+^ T cells as well as an increase in cytotoxic CD4^+^ granzyme B^+^ and CD8^+^ granzyme B^+^ T cells in the combination treatment group (Figure 5C-H). Interestingly, the densities of CD4^+^ and CD4^+^ granzyme B^+^ T cells were higher than those of CD8^+^ and CD8^+^ granzyme B^+^ T cells, suggesting distinct immune responses. Furthermore, we observed the formation of perivascular immune niches, particularly in the combination therapy group, indicating potential remodeling of the tumor vasculature and enhanced immune cell interactions (Figure 5B-H).

Overall, these findings highlight the potential of autophagy inhibition in enhancing the therapeutic effects of anti-PD-1 blockade and low-dose chemotherapy. This represents a promising strategy for improving anti-tumor efficacy and optimizing cancer treatment.

### Inhibiting autophagy activates p53, inducing endothelial cell apoptosis in dysfunctional tumor vessels

Hypoxia and nutrient deprivation are well-known triggers of autophagy, allowing cells to survive and maintain homeostasis under stressful conditions by counteracting apoptosis 22. We hypothesized that TECs, especially those in dysfunctional tumor vessels, utilize autophagy to resist apoptosis induced by hypoxic and nutrient-deprived TMEs, thereby promoting their survival. To investigate this, we employed mIF staining, which revealed that TECs exhibited a high level of autophagy while expressing low levels of cleaved caspase-3, a marker of apoptosis (Figure S5). This suggests that under normal TME conditions, endothelial cells favor autophagy during apoptosis, supporting their survival.

**Figure 6:**
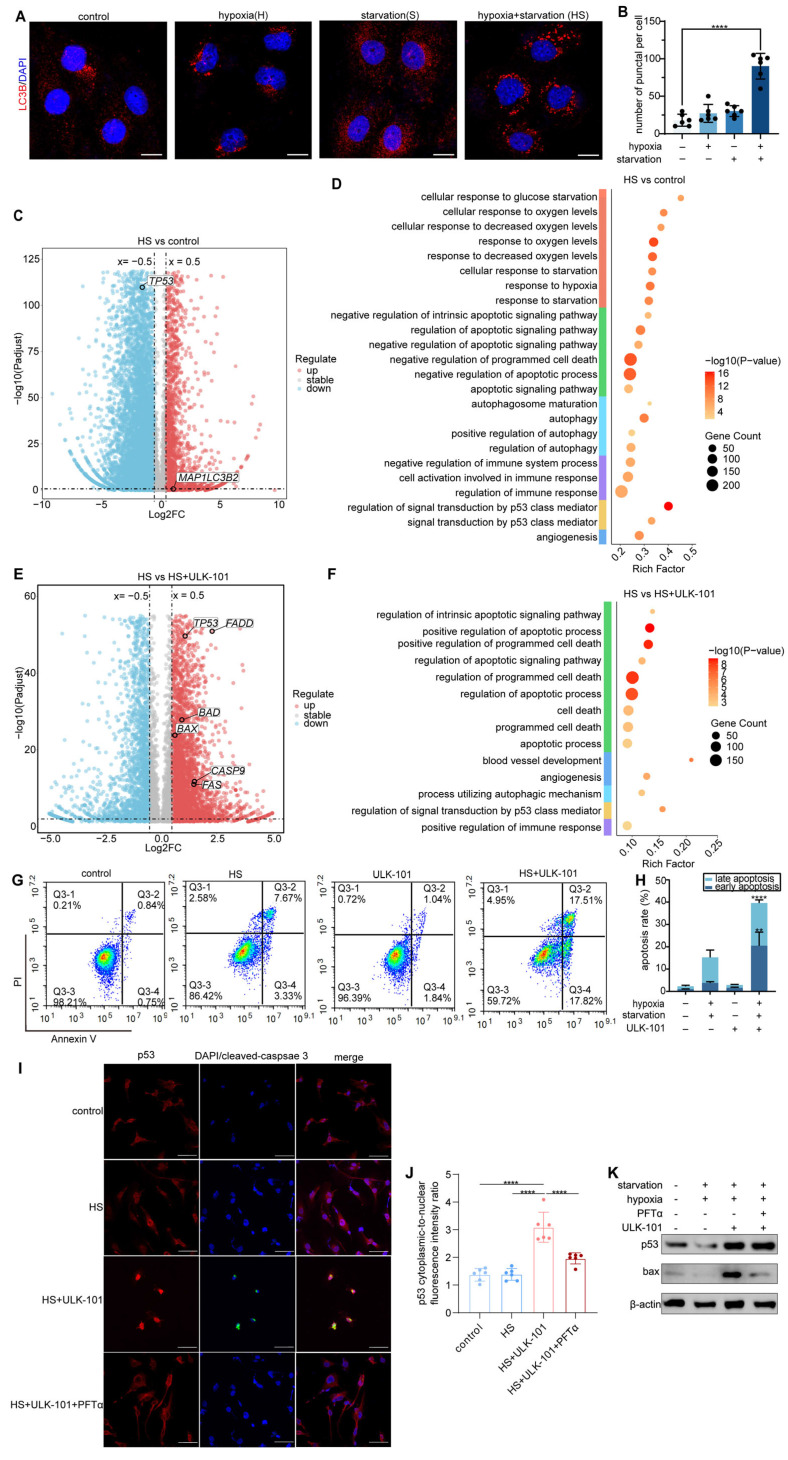
** Inhibition of autophagy activates p53 and induces apoptosis in endothelial cells under hypoxia and nutrient deprivation.** A and B: Immunofluorescent staining of HUVECs cultured under different conditions: control, hypoxia (1% O₂), starvation (glucose and serum starvation in MEM), and combined hypoxia and starvation (HS). LC3B (red) marks autophagic activity, whereas DAPI (blue) stains the nuclei. Quantification of LC3B puncta-positive cells showed a significant increase in autophagy under combined hypoxia and nutrient deprivation compared to that in the control (****P < 0.0001). Scale bar = 50 μm. C: Volcano plot displaying DEGs in HUVECs cultured under hypoxia and nutrient deprivation versus controls. Upregulated genes are highlighted in red, downregulated genes in blue, and stable genes in grey. *MAP1LC3B2* expression was significantly upregulated, indicating elevated autophagic activity. D: GO analysis showing the enriched pathways in HUVECs cultured under hypoxia and nutrient deprivation. E: Volcano plot comparing gene expression in HUVECs treated with hypoxia and nutrient deprivation combined with 4 μmol/L ULK-101 (autophagy inhibitor) versus hypoxia and nutrient deprivation alone. Apoptosis-related genes, including *FADD*,* BAX*,* CASP9*, and* FAS*, were significantly upregulated, while *TP53* expression was restored by ULK-101 treatment. F: GO analysis of HUVECs treated with ULK-101 under hypoxia and nutrient deprivation conditions, showing significant enrichment of apoptosis-related pathways, angiogenesis, and positive regulation of immune responses. G and H: Flow cytometry analysis of apoptosis in HUVECs under control, hypoxia and nutrient deprivation (HS), 4 μmol/L ULK-101, and combined ULK-101 and HS treatments. Apoptosis was measured using Annexin V/PI staining. Quantification (H) showed a significant increase in apoptosis in HUVECs treated with ULK-101 under hypoxic and nutrient-deprived conditions (****P < 0.0001; **P < 0.01). I and J: Immunofluorescence staining of HUVECs treated with control, hypoxia and nutrient deprivation (HS), 4 μmol/L ULK-101, and combined ULK-101 and HS treatments, with and without the p53 inhibitor pifithrin-α (PFTα) HBr (10 μmol/L). P53 (red) and cleaved caspase-3 (green) indicate apoptosis, with DAPI staining of the nuclei (blue). Quantification revealed a significant increase in nuclear p53 translocation after ULK-101 treatment (J). Scale bar = 50 μm. K: Western blot analysis of p53 and BAX protein expression in HUVECs under control, hypoxia and nutrient deprivation (HS), 4 μmol/L ULK-101, and combined treatment with 10 μmol/L PFTα. The 4 μmol/L ULK-101 treatment significantly increases p53 and BAX expression under hypoxia and nutrient deprivation.

To validate this hypothesis, we established an *in vitro* model by culturing human umbilical vein endothelial cells (HUVECs) under hypoxia (1% O₂) combined with glucose and serum deprivation to simulate the severe hypoxic and nutrient-deprived conditions experienced by endothelial cells in dysfunctional tumor vessels. IF staining confirmed that this combined stress induced significant autophagy in HUVECs (Figure 6A-B). Given the difficulty in isolating endothelial cells from dysfunctional tumor vessels *in vivo* owing to the lack of specific biomarkers, this *in vitro* model allowed us to explore how inhibiting autophagy affects endothelial cell survival and the optimization of tumor vasculature and the TiME.

Transcriptomic analysis revealed that hypoxia and nutrient deprivation upregulated the autophagy-associated gene *MAP1LC3B2* (Figure 6C) and downregulated *TP53*. Previous studies suggested that p53 suppression promotes autophagy through a protective mechanism against apoptosis 23. Gene enrichment analysis of HUVECs under these conditions highlighted the activation of pathways related to hypoxia, starvation, and autophagy, as well as the negative regulation of apoptotic and immune pathways (Figure 6D). However, when autophagy was inhibited by ULK-101, apoptosis-related pathways were significantly activated, along with pathways related to angiogenesis and positive regulation of immune responses (Figure 6F). Volcano plot analysis showed the upregulation of apoptosis-related genes, such as *FADD*, *BAX*, *CASP9*, and *FAS*, under ULK-101 treatment (Figure 6E). In addition, *TP53* expression was restored in the presence of ULK-101(Figure 6E).

Flow cytometry confirmed the inhibition of autophagy in HUVECs cultured under hypoxic conditions and nutrient deprivation-induced apoptosis (Figure 6G-H). These findings suggest that autophagy inhibition in endothelial cells from dysfunctional tumor vessels leads to apoptosis, selective pruning of these cells, and optimization of the structure and function of the tumor vasculature. Transcriptomic analysis indicated that this process is mediated by the activation of p53, a key regulator of both autophagy and apoptosis (Figure 6E-F).

To further confirm the involvement of p53 in this mechanism, we co-treated HUVECs with ULK-101 and pifithrin-α (PFTα), a p53 inhibitor, under hypoxia and nutrient deprivation. IF and western blot analyzes demonstrated that autophagy inhibition activated p53, which was translocated to the nucleus and induced apoptosis in HUVECs. Co-treatment with PFTα reversed the apoptosis induced by autophagy inhibition (Figure 6I-J). Additionally, western blot analysis showed that ULK-101 treatment upregulated both p53 and the pro-apoptotic protein BAX, while PFTα co-treatment reversed this effect (Figure 6K).

Collectively, these results demonstrated that endothelial cells in dysfunctional tumor vessels depend on autophagy to survive under severely hypoxic and nutrient-deprived conditions, thereby preventing apoptosis. When autophagy is inhibited, p53 is activated, triggering apoptosis and selective pruning of dysfunctional tumor vessels, thereby optimizing the vasculature.

## Discussion

Optimizing tumor vasculature is crucial in cancer therapy because it profoundly influences tumor progression, metastasis, and treatment response 4. However, effective pharmaceutical agents targeting tumor vasculature remain limited in clinical practice. In this study, we investigated the selective elimination of dysfunctional tumor vascular sprouts as a strategy to improve the abnormal structure and impaired functionality of tumor vasculature. Additionally, we sought to characterize the unique features of endothelial cells within dysfunctional tumor vessels to inform vascular optimization strategies.

Autophagy, a conserved metabolic process in eukaryotic cells, has also been observed in TECs 16. However, its precise role and impact on the tumor vasculature, particularly in dysfunctional vessels, remain poorly understood. Given the elevated levels of hypoxia and nutrient deprivation within dysfunctional tumor vessels due to poor blood flow, both of which are key inducers of autophagy, we hypothesized that autophagy would be upregulated in TECs within these vessels. Therefore, targeting endothelial cell autophagy may have the potential to optimize aberrant tumor vasculature. To test this hypothesis, we used clinical tumor tissue samples, scRNA sequencing data, a zebrafish xenograft model, and a mouse melanoma lung metastasis model. Our findings confirmed that autophagy is prevalent in TECs and is particularly elevated in dysfunctional endothelial cells.

To further explore how autophagy inhibition contributes to the optimization of the abnormal tumor vasculature, we employed our previously reported tumor vessel visualization platform 3. Using this platform, we demonstrated that autophagy inhibitors selectively prune dysfunctional tumor vascular sprouts, leading to significant improvements in vascular structure and functionality. To confirm that this vascular optimization was mediated by the inhibition of endothelial cell autophagy, we generated a conditional endothelial cell-specific *Atg7* knockout mouse model. Prior research by Maes *et al.* demonstrated that tamoxifen-induced *Atg5* knockout prior to tumor implantation leads to increased disorganization of the tumor vasculature 24. Thus, we induced *Atg7* knockout with tamoxifen post-tumor transplantation, and our results demonstrated that suppressing endothelial cell autophagy after vascular network formation promoted improved vascular architecture and function.

We also observed significant enhancements in the TiME, including increased infiltration of CD4^+^ and CD8^+^ T cells and formation of perivascular immune niches following autophagy inhibition. Our findings align with those of Verhoeven *et al.*, who identified autophagy as a key anti-inflammatory mechanism within the tumor vasculature that suppresses melanoma immunity 16. Our investigation of the combination of autophagy inhibitors with anti-PD-1 therapy or low-dose chemotherapy revealed that autophagy inhibition significantly enhanced chemotherapy efficacy. While combining autophagy inhibitors with PD-1 blockade also improved the anti-tumor effects compared to PD-1 therapy alone, the synergistic effect was moderate relative to that observed with chemotherapy. Several factors may contribute to this: firstly, the relatively low immunogenicity of CT26 cells and their further compromised immunogenicity in subcutaneous tumors could explain the limited response to immunotherapy 25, 26. Secondly, the sequence of administration of anti-PD-1 and autophagy inhibitors may also affect the effectiveness of combination therapy. Verhoeven *et al.* suggested that autophagy inhibition primarily sustains, rather than enhances, the immune response in tumor immunotherapy 16. In our study, we observed that autophagy inhibition significantly activated CD4^+^ granzyme B^+^ T cells more than CD8^+^ granzyme B^+^ T cells. However, the specific role of CD4^+^ granzyme B^+^ T cells in tumor immunity remains unclear and warrants further investigation 27. Additionally, autophagy inhibitors may exert broad effects at the whole-tumor level, thereby influencing various aspects of tumor immunity. Therefore, further studies are required to assess the status of immune cell populations, including immunosuppressive cells. In the context of low-dose chemotherapy, autophagy inhibitors may potentiate the anti-cancer effects via mechanisms other than immune modulation. The anti-metabolite 5-FU disrupts tumor cell proliferation by interfering with DNA and RNA synthesis 28. When combined with autophagy inhibition, which compromises tumor cell survival, the two therapies act through complementary pathways to produce a more robust anti-tumor effect.

Furthermore, in *Atg7*^iECKO^ melanoma lung metastasis and subcutaneous tumor models, we did not observe significant inhibition of tumor progression or development following endothelial cell-specific* Atg7* gene deletion. Similarly, treatment with the autophagy inhibitor SBI-0206965 as a monotherapy showed limited anti-tumor effects in the CT26 subcutaneous tumor model, which may be related to the systemic administration route. These findings suggest that the inhibition of endothelial cell autophagy alone may not be sufficient to halt tumor progression or development. However, given its role in optimizing tumor vasculature and the TiME, the inhibition of autophagy could still serve as an effective tumor sensitization strategy when combined with other therapies.

Given the challenges of isolating endothelial cells from dysfunctional tumor vessels owing to the lack of definitive biomarkers, we used *in vitro* models of HUVECs cultured under hypoxic and nutrient-deprived conditions to simulate the microenvironment of dysfunctional tumor vessels. Our study demonstrated that hypoxic and nutrient-deprived conditions induce autophagy in HUVECs, suggesting that autophagy serves as a critical survival mechanism for dysfunctional endothelial cells in the TME. Furthermore, inhibiting autophagy activates p53, leading to the apoptosis of dysfunctional endothelial cells and selective pruning of abnormal tumor vessels. These findings suggest that further research on the role of p53 activation in dysfunctional TECs may provide valuable insights into tumor vascular remodeling and optimization.

In this study, we used the ULK1 inhibitors SBI-0206955, ULK-101, and MRT68921 to inhibit autophagy. ULK1 is a key kinase that initiates autophagy, and its inhibition effectively blocks autophagy. In contrast, other autophagy inhibitors such as chloroquine lack specificity 24. Our findings suggest that targeting ULK1 is a promising strategy for optimizing aberrant tumor vasculature, as demonstrated in both mouse and zebrafish models. ULK1 inhibitors also show potential for synergistically enhancing the effects of chemotherapy and immunotherapy. However, ULK1 inhibitors are still under development, and clinically available options remain limited 29. As research progresses, the value of ULK1 inhibitors in targeting the tumor vasculature and enhancing cancer therapy will become clearer.

In summary, our study offers several key insights. Endothelial cells within dysfunctional tumor vasculature demonstrate elevated autophagy, functioning as a protective mechanism within the hypoxic and nutrient-deprived TME. Autophagy inhibition activates p53, leading to the apoptosis of these dysfunctional endothelial cells. Targeted inhibition of endothelial autophagy selectively prunes aberrant vascular sprouts, resulting in the remodeling and functional enhancement of tumor vasculature. Additionally, the inhibition of autophagy improves the TiME by facilitating the formation of perivascular immune niches that support immune cell infiltration and activation. The combination of autophagy inhibitors with chemotherapy and immunotherapy has emerged as a promising therapeutic approach to boost anti-tumor efficacy.

## Materials and Methods

### Patient and sample collection

Human tissue samples, including samples of GBM, hepatocellular carcinoma, NSCLC, ICC, colon cancer, GC, and adjacent normal tissues, were obtained from patients who underwent surgery at West China Hospital, Sichuan University. All specimens were pathologically confirmed. This study was approved by the Ethics Committee of West China Hospital, Sichuan University, and written informed consent was obtained from all participants.

### Bioinformatics analysis methods

We conducted a comprehensive analysis of autophagy-related gene expression in normal and TECs using single-cell RNA sequencing (sc-RNA-seq) data from a previously published NSCLC study 18. The dataset obtained from the NCBI Gene Expression Omnibus (GSE131907) included endothelial cells isolated from both normal lung tissues and NSCLC samples. Raw unique molecular identifier count matrices and corresponding cell annotation files were preprocessed using Seurat (v4.3.0.1) in R (v4.3.1). Dimensionality reduction was performed using principal component analysis. Clustering was conducted using the FindNeighbors and FindClusters functions, followed by visualisation with Uniform Manifold Approximation and Projection and t-distributed stochastic neighbour embedding (t-SNE).

To analyze autophagy-related gene expression, we curated a set of autophagy-related genes based on the literature and calculated their detection frequencies and mean expression levels. Scatter plots were generated using ggplot2, where the point size and colour represent the detection percentages and mean expression, respectively. The autophagy score for each cell was computed using the AddModuleScore function in Seurat based on 245 autophagy-related genes. Violin plots were generated to display the distribution of autophagy scores between normal and TECs.

For functional enrichment analysis, TECs were stratified into high- and low-autophagy score groups using a threshold of 0.15, which was chosen based on the distribution of autophagy scores. DEGs between these groups were identified using the FindMarkers function, with thresholds of *P* < 0.05 and an average log2 fold change (avg_log2FC) > 0.5. Gene Ontology (GO) term enrichment analysis was performed on the DEGs using the enrichGO function from clusterProfiler (v4.10.0), with the Benjamini-Hochberg method applied to correct for multiple comparisons (*p*.adjust < 0.05).

Additionally, TECs were stratified based on HIF-1α expression. Autophagy-related gene expression, including detection frequencies and mean expression levels, was assessed in HIF-1α-positive and HIF-1α-negative groups. Scatter plots were generated using ggplot2 to visualise differences.

### Zebrafish xenograft angiogenesis and analysis

Zebrafish husbandry, zebrafish functional xenograft vasculature platform (zFXVP) construction, agent administration, and tumor vasculature imaging and quantification were conducted following established protocols described in our previous study 3. Zebrafish were maintained and bred under standard conditions. All zebrafish experiments were approved by the Animal Ethics Committee of West China Hospital, Sichuan University and conducted in compliance with the institutional guidelines for the care and use of laboratory animals.

Transgenic zebrafish xenograft angiogenesis models (flk-GFP/LC3-mcherry; flk:eGFP; flk:mCherry; flk:eGFP/ Gata1:dsRed) were used as previously described 3. *In vivo* 3D imaging of the tumor vasculature was performed using confocal fluorescence microscopy (ZEISS LSM 880 + Airyscan; Carl Zeiss, Germany), with a sample size of 20 zebrafish per experimental condition. The distribution of LC3-mCherry aggregates within endothelial cells was quantitatively assessed using ImageJ software (v1.53).

### Evaluation of tumor vasculature structure and function optimization by autophagy inhibitors in zebrafish xenograft models

Transgenic zebrafish (flk:eGFP; flk:mCherry; flk:eGFP/Gata1:dsRed) with comparable tumor sizes were randomly assigned to either the autophagy inhibitor treatment group or the control group. The treatment group received chloroquine (50 μmol/L), ULK-101 (4 μmol/L), and MRT68921 (50 μmol/L) (all purchased from Selleck, USA), which were diluted to the required concentrations in dimethyl sulfoxide (DMSO). The control group was administered an equivalent dose of DMSO. Both drugs and DMSO were replenished daily throughout the experimental period. The tumor vasculature was visualized *in vivo* after 5 d of treatment using a confocal fluorescence microscope (ZEISS LSM 880 + Airyscan; Carl Zeiss, Germany). Image analysis and quantification of the tumor vasculature were performed using ImageJ software (v1.53).

### Injection of FITC-dextran into zebrafish intersegmental vessels

Tumor-bearing transgenic zebrafish (flk:mCherry) were anaesthetized before injecting FITC-dextran (molecular weight 20kDa; Yeasen, China) into the intersegmental vessels near the tail fin. Within 10 min post-injection, FITC-dextran distribution was observed near the heart via fluorescence microscopy (Olympus BX53; Tokyo, Japan), allowing sufficient time for circulation. Imaging and photography were performed using a confocal fluorescence microscope (ZEISS LSM 880 + Airyscan; Carl Zeiss, Germany) to capture the distribution of FITC-dextran within the tumor vasculature. Image analysis and quantification of the tumor vasculature were performed using ImageJ software (v1.53).

### Construction of mouse B16-F10 melanoma lung metastasis model for visualization of autophagy-positive endothelial cells in tumor vasculature

To visualize autophagy-positive endothelial cells within the tumor vasculature, a B16-F10 melanoma lung metastasis mouse model was established using C57BL/6 mice (*n* = 5). All animal experiments were approved by the Animal Ethics Committee of West China Hospital, Sichuan University and conducted in accordance with the institutional guidelines for animal welfare. B16-F10 melanoma cells (50,000 cells/mouse) were injected intravenously into the tail vein.

The tumor vasculature was assessed 12 d post-injection, at which time the neo vasculature developed in the lungs. To evaluate blood perfusion, FITC-dextran (10 mg/mL, molecular weight 2000 kDa; Sigma-Aldrich, USA) was administered intravenously (100 μL) 30 min prior to euthanasia. After FITC-dextran injection, the mice were transcardially perfused with 20 mL of PBS, followed by 20 mL of 4% paraformaldehyde (PFA) to eliminate residual dextran and fix the tissues. Tumor tissues were harvested and subjected to immunofluorescence staining. Tissues were incubated overnight at 4 °C with anti-CD31 (AF594-conjugated, 1:200 dilution; Abcam, UK) and anti-LC3B (AF633-conjugated, 1:200 dilution; Abcam, UK) antibodies, followed by three washes with PBS. Slides were mounted using 80% glycerol, and images were captured using a fluorescence microscope (Olympus BX53; Tokyo, Japan).

### Construction of melanoma subcutaneous tumor and lung metastasis models in endothelial cell-specific *Atg7* conditional knockout mice

All animal experiments were conducted according to the guidelines of the Institutional Animal Care and Use Committee guidelines of Sichuan University. Endothelial cell-specific *Atg7* conditional knockout mice (*Atg7*^iECKO^) were generated by breeding VeCadh-creERT2 mice with *Atg7* floxed gene knockout mice. To specifically induce* Atg7* gene knockout in endothelial cells, tamoxifen (Sigma-Aldrich, USA) was administered intraperitoneally at a concentration of 20 mg/mL dissolved in corn oil. Each mouse received tamoxifen at a dose of 2 mg per 20 g body weight once daily for five consecutive days. Control mice, which were *Atg7* floxed but lacked the Cre recombinase transgene, received equivalent volumes of corn oil without tamoxifen.

To establish the subcutaneous melanoma model, B16-F10 melanoma cells in the logarithmic growth phase were injected subcutaneously into the left hind flank of *Atg7*^iECKO^ and control mice. Each mouse was administered 5 × 10^5^ B16-F10 cells suspended in PBS. To establish a pulmonary metastasis model, B16-F10 cells were intravenously injected into the tail vein. Each mouse was injected with approximately 1 × 10^6^ B16-F10 cells.

### Establishment of the CT26 subcutaneous tumor model and administration of SBI-0206965 with 5-FU or anti-Mouse PD-1 (CD279)

To establish a murine colon cancer subcutaneous tumor model, CT26 cells in the logarithmic growth phase were injected subcutaneously into the left hind flank of BALB/c mice at a concentration of 5 × 10^5^ cells suspended in PBS.

Seven days post-inoculation, the mice were treated with SBI-0206965 (Selleck, USA) via intraperitoneal injection at a dose of 10 mg/kg once daily for four consecutive days. After this treatment, low-dose 5-FU (Selleck, USA) was administered via tail vein injection at a dose of 5 mg/kg every 3 d. In the alternative treatment group, an anti-mouse PD-1 (CD279) antibody (clone BE0273; Bio X Cell, USA) was administered intraperitoneally at a dose of 5 mg/kg every 3 d. Control mice received an equivalent volume of physiological saline via the same route.

Mice were weighed daily, and the tumor size was measured every 2 d using calipers. Tumor volume was calculated using the following formula: (length × width^^2^)/2. On day 14 post-inoculation, mice were euthanized, and tumors were harvested for subsequent multiple immunofluorescence analyzes.

### Multiple immunofluorescence

Tissue slides were baked overnight at 65 °C. Sections were then de-paraffinized and rehydrated using a series of graded solutions as follows: xylene I for 10 min, xylene II for 8 min, and xylene III for 8 min. Following deparaffinization, the sections were rehydrated by immersion in absolute ethanol for 5 min, 90% ethanol for 3 min, 80% ethanol for 3 min, and 70% ethanol for 3 min and finally rinsed three times in distilled water for 3 min. Sections were then immersed in PBS for 3 min, and this was repeated three times to prepare for antigen retrieval.

Antigen retrieval was performed using Tris-ethylenediaminetetraacetic acid buffer (pH 9.0). Tissue sections were heated in a microwave for 20 min, followed by cooling to room temperature. Endogenous peroxidase activity was blocked by incubating the sections in 3% hydrogen peroxide (H₂O₂) for 10 min. After washing with PBS, the sections were blocked with goat serum for 30 min at room temperature to reduce nonspecific binding.

Next, the sections were incubated with the following primary antibodies for 1 h at 37 °C in a humidified chamber: granzyme B (1:2,000 dilution; HA500252; HUABIO, China), CD4 (1:1,000 dilution; ab183685; Abcam, China), CD8 (1:300 dilution; GB114196; Servicebio, China), CD31 (1:300 dilution; GB114196; Servicebio, China), α-SMA (1:3,000 dilution; ET1607-53; HUABIO, China), CD3 (1:1,000 dilution; HA720082; HUABIO, China), CA-IX (1:1,000 dilution; ET1701-51; HUABIO, China), Beclin-1 (1:1,000 dilution; HA721216; HUABIO, China), p62 (1:1,000 dilution; HA721171; HUABIO, China), and LC3B (1:1,000 dilution; ET1701-65; HUABIO, China).

Following incubation with primary antibodies, the sections were processed using the IRISKit® HyperView mIF Kit (MH010101; LUMINIRIS, China), according to the manufacturer's instructions for tyramide signal amplification (TSA) staining. This kit allows sequential and multiplex staining of multiple targets within a single tissue section while preserving antigen integrity.

After completion of the staining protocol, the slides were counterstained (if applicable) and mounted with 80% glycerol. Fluorescence images were captured using the VS200 system (Olympus, Tokyo, Japan). Multi-round staining images were merged using ImageJ software (v1.53). The acquired images were subsequently analyzed using QuPath software (v0.4.3) to quantify marker expression and evaluate the spatial distribution of the stained markers within the tissue sections.

### Cell lines and culture

HUVECs were purchased from the American Type Culture Collection (ATCC) and cultured in endothelial cell medium (ECM; ScienCell, USA). CT26 murine colon adenocarcinoma cells (ATCC CRL-2638) and B16-F10 murine melanoma cells (ATCC CRL-6475) were cultured in Roswell Park Memorial Institute (Gibco, USA) 1640 medium supplemented with 10% FBS and penicillin/streptomycin. Cells were cultured in a 5% CO_2_ and 95% O_2_ environment at 37 °C.

### Hypoxia, glucose, and serum deprivation-induced culture of HUVECs and treatment with autophagy and p53 inhibitors

To simulate hypoxic and nutrient-deprived conditions, HUVECs were cultured in either an anaerobic modular incubator (95% N₂, 5% CO₂, 1% O₂) or a normoxic incubator. Cells were divided and maintained in either standard ECM or serum- and glucose-free minimum essential medium (MEM; Thermo Fisher Scientific, USA) for 48 h. During this period, cells were co-treated with the autophagy inhibitor ULK-101 (4 μmol/L) and the p53 inhibitor pifithrin-α (PFTα) HBr (10 μmol/L) under hypoxic, glucose-deprived, and serum-deprived conditions for 48 h.

### Flow cytometric analysis

HUVECs cultured under hypoxic and nutrient-deprived conditions for 48 h in the presence of the autophagy inhibitor ULK-101 (4 mmol/L) were dissociated using trypsin without EDTA. Cells were centrifuged at 2,000 rpm for 5 min, washed twice with PBS (2,000 rpm for 5 min each), and resuspended in binding buffer at a concentration of 5 × 10^5^ cells. Annexin V-FITC and propidium iodide (PI) were added to the cell suspension, mixed gently, and incubated at room temperature in the dark for 10 min. Samples were immediately analyzed using a FACSCanto flow cytometer (BD Biosciences).

### Cell immunofluorescence

HUVECs grown on coverslips were fixed in ice-cold methanol for 10 min and blocked with 1% BSA in PBST (PBS + 0.1% Tween 20) for 60 min. Cells were incubated overnight at 4 °C with the following primary antibodies: anti-LC3B (1:100; ET1701-65; Abcam, China), anti-p53 (1:500; EM20603; HUABIO, China), or anti-cleaved caspase-3 (1:5,000; Cat. no. 9579S; Cell Signalling Technology).

Following primary antibody incubation, the cells were processed using the IRISKit® HyperView mIF Kit (MH010101; LUMINIRIS, China) following the manufacturer's protocol for TSA staining. After washing three times with PBS, the cells were counterstained with 4',6-diamidino-2-phenylindole (DAPI; Abcam) for 5 min and mounted with 80% glycerol.

Fluorescent images were captured using a spinning disc confocal microscope (Olympus, Tokyo, Japan). Quantitative analysis of autophagic puncta and nuclear and cytoplasmic fluorescence intensity of p53 was performed using QuPath software (v0.4.3).

### Transcriptome sequencing of HUVECs

Total RNA was extracted from cultured HUVECs using TRIzol® reagent (Thermo Fisher Scientific, USA) according to the manufacturer's protocol. RNA quality was assessed using a 5300 Bioanalyzer (Agilent, USA), and RNA concentration was measured using an ND-2000 spectrophotometre (NanoDrop Technologies, USA). Only high-quality RNA samples meeting the following criteria were used for library construction: OD260/280 = 1.8-2.2, OD260/230 ≥ 2.0, RNA Quality Number (RQN) ≥ 6.5, 28S:18S ≥ 1.0, and total RNA ≥ 1 μg.

RNA purification, reverse transcription, library construction, and sequencing were performed by Shanghai Majorbio Bio-Pharm Biotechnology Co., Ltd. (Shanghai, China). Libraries were prepared using the Illumina® Stranded mRNA Prep, Ligation kit (San Diego, CA, USA) following the manufacturer's instructions, with 1 μg of total RNA per sample. Poly(A)+ mRNA was isolated using fragmented oligo (dT) beads and used for double-stranded cDNA synthesis using the SuperScript double-stranded cDNA synthesis kit (Invitrogen, CA, USA). cDNA fragments of approximately 300 bp were size-selected using a 2% low-range ultra-agarose gel, followed by PCR amplification using Phusion DNA polymerase (NEB, USA) for 15 cycles. Libraries were quantified using Qubit 4.0 (Thermo Fisher Scientific, USA) and sequenced on the NovaSeq X Plus platform (PE150) or DNBSEQ-T7 platform (PE150), according to the respective kit protocols.

Raw paired-end reads were quality-controlled and trimmed using Fastp with the default settings. Clean reads were then aligned to the reference genome using HISAT2 in the orientation mode. Mapped reads were assembled for each sample using StringTie following a reference-based approach.

Transcript expression levels were quantified using the transcripts per million method, and gene abundance was calculated using RSEM. Differential expression analysis was performed using DEGseq, with DEGs defined by |log2FC| ≥ 0.5 and and FDR < 0.001 (for DEGseq). Functional enrichment analysis was performed to identify significantly enriched biological processes and pathways using GOatools and Python scipy packages. GO terms related to biological processes, cellular components, and molecular functions were considered significant, with a Bonferroni-corrected* P*-value of < 0.05.

Volcano plots were generated to visually display DEGs using the ggplot2 package in R. The x-axis represents the log2 fold change (log2FC) in gene expression, whereas the y-axis represents the -log10 adjusted *P*-value. DEGs with significantly increased or decreased expression are highlighted in red and blue to indicate upregulated and downregulated genes, respectively. Stable genes are marked in grey font. Specific genes of interest, such as *TP53* and *MAP1LC3B*, were labelled in the plots for further analysis.

### Western blotting

HUVECs were lysed using RIPA buffer supplemented with 1 mM PMSF (Yeasen, China). Protein concentration was determined, and equal amounts of protein (30 μg per sample) were separated by sodium dodecyl sulfate-polyacrylamide gel electrophoresis (SDS-PAGE) on 10% polyacrylamide gels. Proteins were then transferred onto polyvinylidene difluoride (PVDF) membranes (Bio-Rad, Hercules, CA, USA) using a wet transfer method.

Following transfer, the membranes were blocked for 1 h at room temperature with 5% nonfat milk dissolved in TBS containing 0.1% Tween-20 (TBST) to prevent nonspecific binding. Membranes were then incubated overnight at 4 °C with the following primary antibodies: anti-p53 (1:500; EM20603; HUABIO, China), anti-BAX (1:1,000; Cat. No. 2772T; Cell Signaling Technology, USA), and anti-β-catenin (1:5,000; GB150016-100; Servicebio, China).

After incubation with the primary antibody, the membranes were washed with TBST and incubated with horseradish peroxidase (HRP)-conjugated secondary antibodies for 1 h at room temperature. Membranes were re-washed with TBST, and protein bands were visualized using NcmECL SuperUltra Reagent (NCM Biotech, China). Chemiluminescent signals were detected using an eBLOT Western Blot Imaging System.

### Statistical analysis

Statistical analysis was conducted using GraphPad Prism software (version 8.0), with a minimum of three technical replicates per sample. *P* values were generated using Student's t-test, one-way ANOVA with Tukey's post-hoc test, or two-way ANOVA with Bonferroni post-tests, with significance set at *P* < 0.05.

## Supplementary Material

Supplementary figures.

## Abbreviations

TME: tumor microenvironment
PD-1: programmed cell death protein
HCC: hepatocellular carcinoma
NSCLC: non-small cell lung cancer
GBM: glioblastoma
GC: gastric cancer
CRC: colorectal cancer
mIF: multiplex immunofluorescence
ICC: intrahepatic cholangiocarcinoma
scRNA-seq: single-cell RNA sequencing
IF: immunofluorescence
HIF-1α: hypoxia-inducible factor 1-alpha
HUVEC: human umbilical vein endothelial cells
VEGF: vascular endothelial growth factor
VEGFR: vascular endothelial growth factor receptor
TEC: tumor endothelial cell
NEC: normal endothelial cell
CA-IX: carbonic anhydrase IX
FITC: fluorescein isothiocyanate
TiME: tumor immune microenvironment
TSA: tyramide signal amplification
