# Supplementary material for: Inhibiting autophagy selectively prunes dysfunctional tumor vessels and optimizes the tumor immune microenvironment
Source: Theranostics. 2025 Jan 1;15(1):258–76. doi: 10.7150/thno.98285 (PMC11667230; doi:10.7150/thno.98285)
Supplement: Supplementary file 1 — Supplementary figures. [file thnov15p0258s1.pdf]

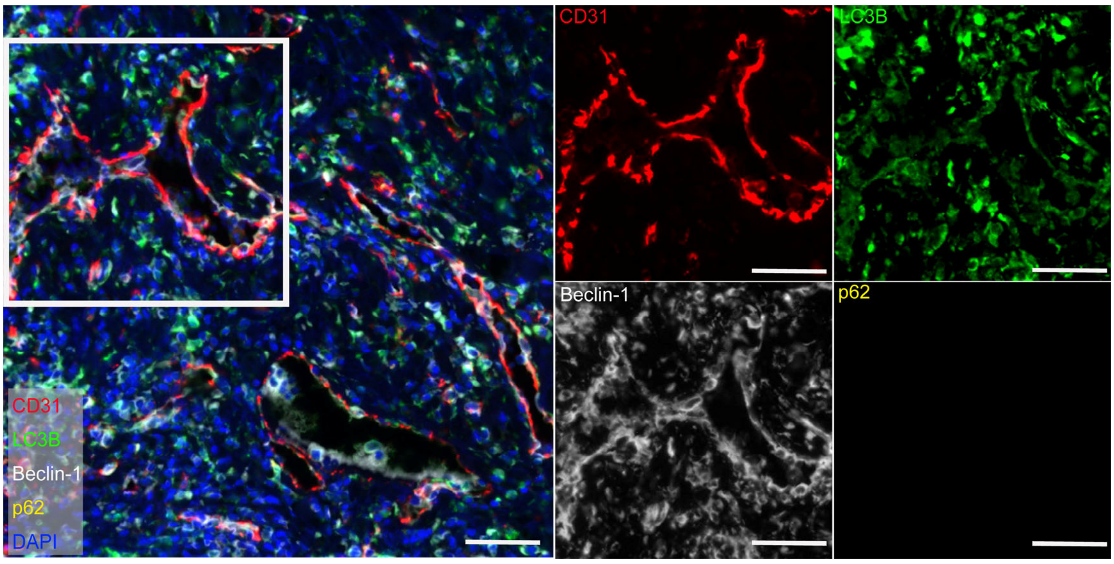

Figure S1. Multiplex immunofluorescence reveals autophagy activity in tumor vasculature.

Multiplex immunofluorescence analysis of autophagic activity in the tumor vasculature of ICC. Scale bar = 50 μm.

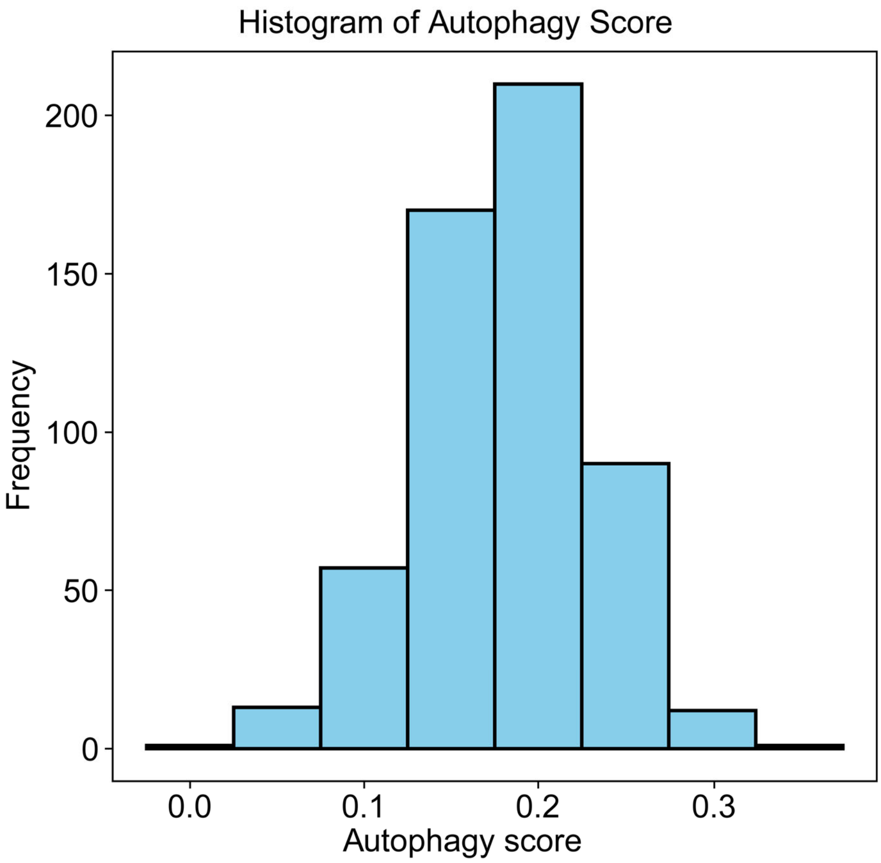

Figure S2. Distribution of tumor endothelial autophagy scores.

Histogram representing the distribution of autophagy scores for TECs assessed using the AddModuleScore function in Seurat for 245 autophagy-related genes. A cutoff of

0.15 distinguishes cells with high- versus low-autophagy scores.

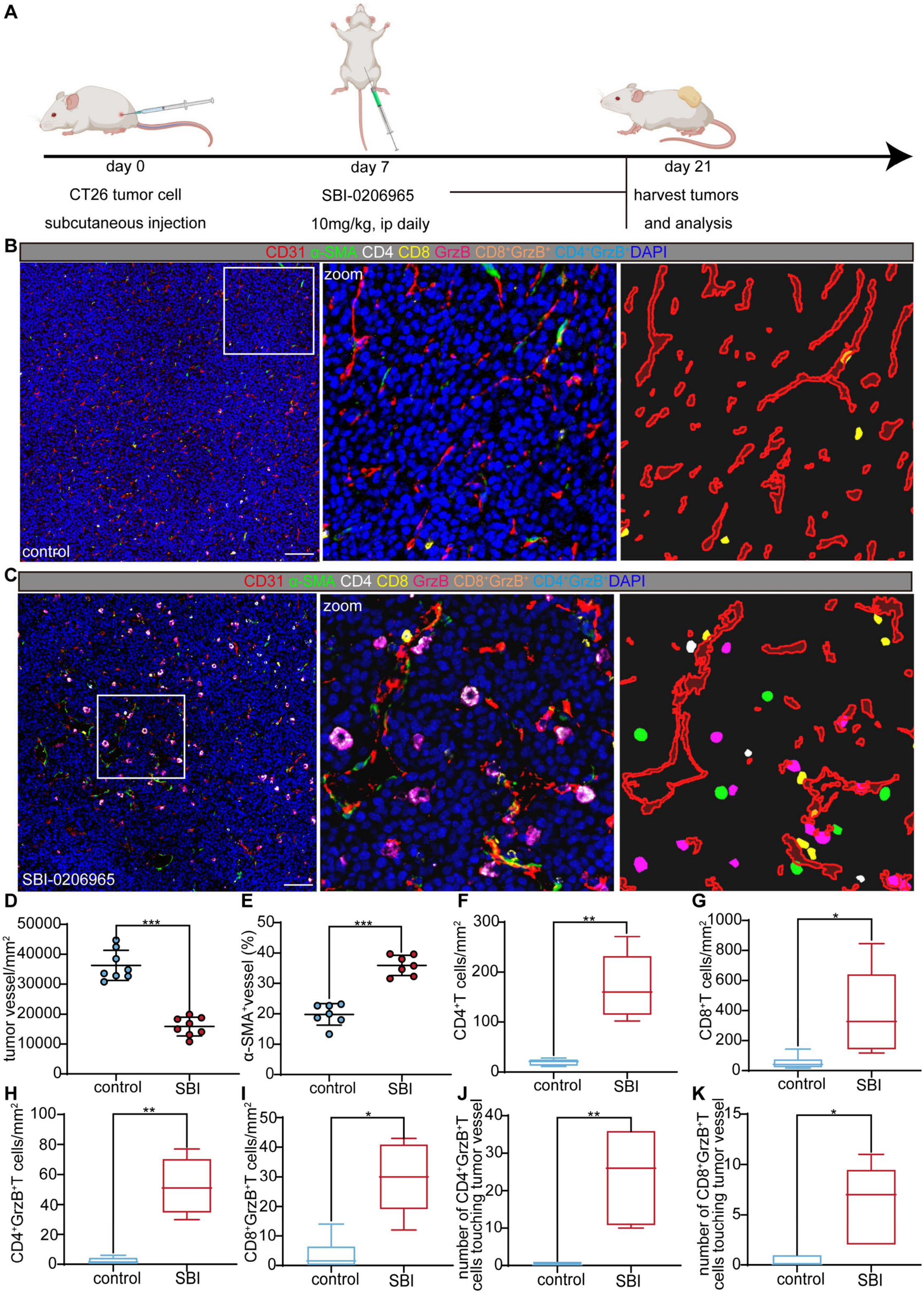

Figure S3. Autophagy inhibitor SBI-0206965 enhances immune infiltration and remodels the vascular structure in the CT26 tumor model.

A: Schematic representation of the experimental timeline for the CT26 murine subcutaneous tumor model, illustrating the initiation of autophagy inhibition by SBI-0206965 (10 mg/kg, administered intraperitoneally daily), beginning on day 7. Tumors

were excised on day 21 for further analysis.

B: Representative multi-immunofluorescence (mIF) images of the tumor immune microenvironment (TiME) in control mice, showing vascular networks (CD31, red) and pericytes (α-SMA, green), along with helper T cells (CD4, white), cytotoxic T cells (CD8, yellow), and granzyme B (GrzB, magenta). Nuclei were stained with DAPI (blue). The zoomed-in area highlights the interaction between vasculature and immune cells within the TiME of the control group. Scale bar = 100 μm.

C: mIF staining of the TiME in SBI-0206965-treated mice, showing an increased density of functional T cells ($CD4^+$ granzyme B^+ and $CD8^+$ granzyme B^+) around mature vessels with large lumens and α-SMA^+ coverage. The zoomed-in area shows the formation of the perivascular immune niche. Scale bar = 100 μm.

D and E: Quantitative analysis of tumor vessel density (D) and the proportion of functional tumor vessels (α-SMA^+ $CD31^+$ vessels) (E). Data are presented as the mean ± SD.

F–I: Quantitative analysis of $CD4^+$ (F), $CD8^+$ (G), $CD4^+$ granzyme B^+ (H), and $CD8^+$ granzyme B^+ T cell (I) densities. Box plots display the maximum and minimum values, medians, and 25/75 percentiles.

J and K: Quantification of $CD4^+$ granzyme B^+ T cells (J) and $CD8^+$ granzyme B^+ T cells (K) within a 10-μm radius around tumor blood vessels. Box plots display the maximum and minimum values, medians, and 25/75 percentiles.

P values were calculated using Student's t-test. $^*P < 0.05$, $^{**}P < 0.01$, $^{***}P < 0.001$; $n = 7$ mice per group.

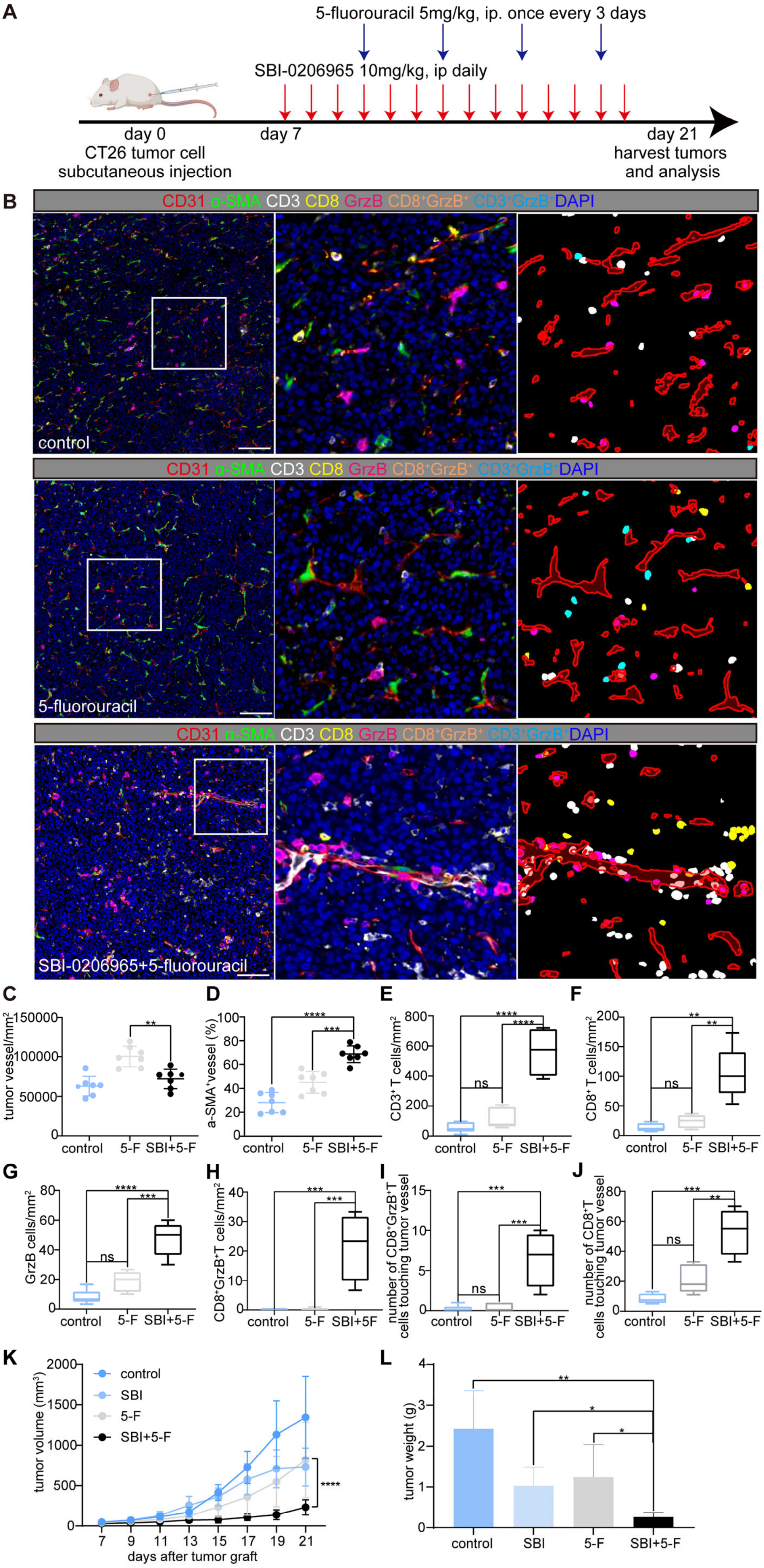

A
5-fluorouracil 5mg/kg, ip. once every 3 days
SBI-0206965 10mg/kg, ip daily
day 0
CT26 tumor cell
subcutaneous injection
day 7
day 21
harvest tumors
and analysis
B
CD31 α-SMA CD3 CD8 GrzB CD8⁺GrzB⁺ CD3⁺GrzB⁺ DAPI
control
5-fluorouracil
SBI-0206965+5-fluorouracil
C
tumor vessel/mm²
control 5-F SBI+5-F
D
a-SMA⁺vessel (%)
E
CD3⁺ T cells/mm²
F
CD8⁺ T cells/mm²
G
GrzB cells/mm²
H
CD8⁺GrzB⁺T cells/mm²
I
number of CD8⁺GrzB⁺T cells touching tumor vessel
J
number of CD8⁺T cells touching tumor vessel
K
tumor volume (mm³)
days after tumor graft
control
SBI
5-F
SBI+5-F
L
tumor weight (g)
control SBI 5-F SBI+5-F

Figure S4. Autophagy inhibitor SBI-0206965 synergizes to enhance the anti-tumor effect of low-dose 5-FU.

A: Schematic representation of the experimental timeline for the CT26 subcutaneous tumor model treated with SBI-0206965 and 5-FU. SBI-0206965 (10 mg/kg, intraperitoneally) was administered daily starting on day 7 after tumor cell injection. 5-FU (5 mg/kg, intraperitoneally) was administered once every 3 d. Tumors were harvested on day 21 for further analysis.

B: Representative mIF images showing TiME in control, 5-FU-treated, and combination-treated (SBI-0206965 + 5-FU) groups. The vascular network was visualized using CD31 (red) and α-SMA (green), while immune cell markers included CD3 (T cells, white), CD8 (cytotoxic T cells, yellow), granzyme B (GrzB, magenta), $CD8^+$ $GrzB^+$ (pink), and $CD3^+$ $GrzB^+$ (cyan). Nuclei were stained with DAPI (blue). The right panels show zoomed-in images highlighting the interactions between immune cells and tumor vasculature. Scale bar = 100 μm.

C and D: Quantification of tumor vessel density (C) and the proportion of functional tumor vessels (α-SMA^+ $CD31^+$ vessels) (D). Data are presented as the mean ± SD.

E and F: Quantification of $CD3^+$ T cell density (E) and $CD8^+$ T cell density (F) in the TME across treatment groups. Data are shown as box plots with minimum, maximum, median, and 25/75 percentiles.

G and H: Quantification of granzyme B^+ ($GrzB^+$) cells (G) and $CD8^+$ $GrzB^+$ T cells (H) under different treatment conditions. Data are presented as box plots.

I and J: Quantification of $CD8^+$ $GrzB^+$ T cells (I) and $CD8^+$ T cells (J) within a 10-μm radius around tumor blood vessels. Box plots show the minimum, maximum, median, and 25/75 percentiles.

K: Tumor volume measured over the course of treatment showing the effects of SBI-0206965, 5-FU, and their combination on tumor growth (n = 7 mice per group). Data are presented as the mean ± SD.

L: Tumor weight measured at the time of sacrifice (day 21) compared with the control, SBI-0206965, 5-FU, and combination treatment groups. Data are presented as the mean ± SD (n = 7 mice per group).

P values were calculated using Student's t-test. *$P < 0.05$, **$P < 0.01$, ***$P < 0.001$, ****$P < 0.0001$, ns = not significant.

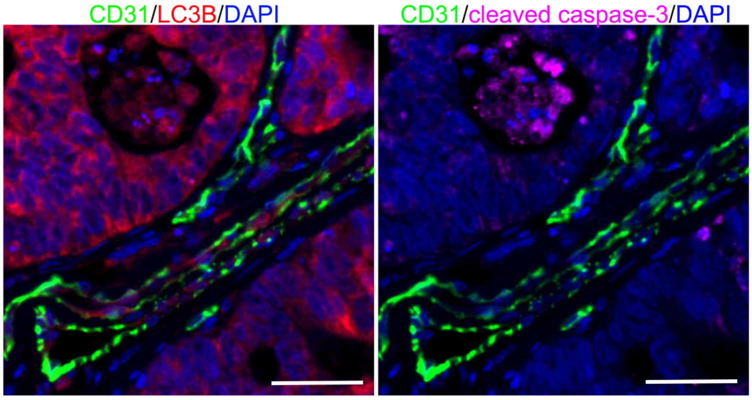

Figure S5. Comparison of autophagy (LC3B) and apoptosis (cleaved caspase-3) in tumor vasculature of CRC specimens.

Representative images of mIF staining in CRC clinical tumor samples showing the expression of CD31 (green) as an endothelial marker, LC3B (red) as an autophagy marker, and cleaved caspase-3 (magenta) as an apoptosis marker. Left panel: Staining with CD31, LC3B, and DAPI (blue) reveals autophagy activity in the tumor vasculature. Right panel: Staining of CD31, cleaved caspase-3, and DAPI shows low levels of apoptosis in endothelial cells within the tumor vasculature. These images demonstrate that autophagy is more prominent than apoptosis in the endothelial cells of the tumor vessels. Scale bar = 50 μm.
